# Large vessels as a tree of transmission lines incorporated in the CircAdapt whole-heart model: A computational tool to examine heart-vessel interaction

**DOI:** 10.1371/journal.pcbi.1007173

**Published:** 2019-07-15

**Authors:** Maarten H. G. Heusinkveld, Wouter Huberts, Joost Lumens, Theo Arts, Tammo Delhaas, Koen D. Reesink

**Affiliations:** 1 Department of Biomedical Engineering, CARIM School for Cardiovascular Diseases, Maastricht University, Maastricht, Netherlands; 2 Department of Biomedical Engineering, Eindhoven University of Technology, Eindhoven, Netherlands; Stanford University, UNITED STATES

## Abstract

We developed a whole-circulation computational model by integrating a transmission line (TL) model describing vascular wave transmission into the established CircAdapt platform of whole-heart mechanics. In the present paper, we verify the numerical framework of our TL model by benchmark comparison to a previously validated pulse wave propagation (PWP) model. Additionally, we showcase the integrated CircAdapt–TL model, which now includes the heart as well as extensive arterial and venous trees with terminal impedances. We present CircAdapt–TL haemodynamics simulations of: 1) a systemic normotensive situation and 2) a systemic hypertensive situation. In the TL–PWP benchmark comparison we found good agreement regarding pressure and flow waveforms (relative errors ≤ 2.9% for pressure, and ≤ 5.6% for flow). CircAdapt–TL simulations reproduced the typically observed haemodynamic changes with hypertension, expressed by increases in mean and pulsatile blood pressures, and increased arterial pulse wave velocity. We observed a change in the timing of pressure augmentation (defined as a late-systolic boost in aortic pressure) from occurring after time of peak systolic pressure in the normotensive situation, to occurring prior to time of peak pressure in the hypertensive situation. The pressure augmentation could not be observed when the systemic circulation was lumped into a (non-linear) three-element windkessel model, instead of using our TL model. Wave intensity analysis at the carotid artery indicated earlier arrival of reflected waves with hypertension as compared to normotension, in good qualitative agreement with findings in patients. In conclusion, we successfully embedded a TL model as a vascular module into the CircAdapt platform. The integrated CircAdapt–TL model allows detailed studies on mechanistic studies on heart-vessel interaction.

## Author summary

Arterial pulse wave characteristics show associations with left ventricular hypertrophy in clinical studies. However, in such studies, measurement and signal processing errors limit assessment of causality between wave characteristics and left ventricular hypertrophy. When validated, a computational model would allow comprehensive causality studies on heart-vessel interaction without such errors. In the present study, we integrated a novel vascular module describing wave transmission in vascular networks into the CircAdapt model of whole-heart mechanics. A benchmark comparison between our vascular module and a previously validated but more complex method, showed good agreement in terms of pressure and flow waveforms. The extended CircAdapt model is now also able to quantitatively describe vascular haemodynamics, including wave dynamics.

## Introduction

The CircAdapt platform, a zero-dimensional whole-heart model developed in our lab, historically focussed on cardiac mechanics. It has been successfully used for simulating haemodynamics during cardiac conductance disorders, valve pathologies, and changes in afterload [1, 2, 3, 4]. Lacking a distributed model of the vascular system, the current CircAdapt model is yet unable to simulate heart-vessel interaction at the level of arterial wave dynamics.

Arterial pulse waves, constituting a component of ventricular afterload, appear to have implications in age-related changes in left ventricular mass, and left ventricular hypertrophy [5, 6]. So-called wave intensity analysis (WIA) allows characterisation of both pulse wave magnitude and propagation direction, thereby requiring synchronous and co-localised measurements of blood pressure and blood flow velocity signals [7]. WIA applied to patient measurement data is sensitive to synchronisation errors and the signal processing characteristics of the measurement devices [8], which hampers or limits detailed studies on heart-vessel interaction, especially concerning causal relationships.

**Fig 1:**
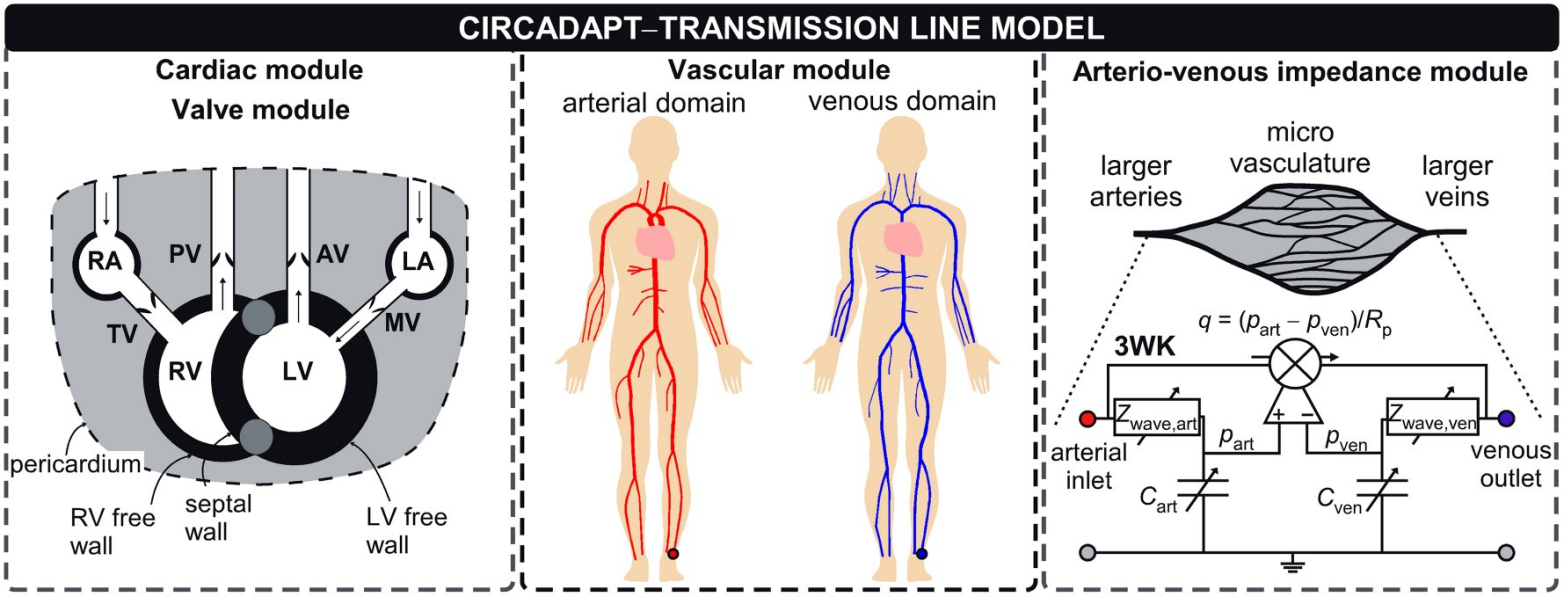
The new vascular module interfaces the existing whole heart-heart mechanics and arterio-venous impedance modules. The CircAdapt–TL model contains the cardiac modules describing whole-heart mechanics, including interventricular interactions, and the systemic and pulmonary circulations [9]. Cardiac valves are modelled as described in Palau-Caballero et al. [1]. The vascular module is described in section ‘Vascular module’. The arterio-venous impedance module, modelling the peripheral circulation using a non-linear three-element windkessel (3WK) was previously developed by Arts et al. [10]. Abbreviations: RA: right atrium, LA: left atrium, TV: tricuspid valve, PV: pulmonary valve, AV: aortic valve, RV: right ventricle, LV: left ventricle, MV: mitral valve, art: arterial, ven: venous, 3WK: non-linear three-element windkessel model. Symbols: *R*_p_: peripheral resistance, *Z*_wave_: wave impedance, *C*: compliance.

Computational models of whole-circulation mechanics, such as CircAdapt, allow for well-controlled simulations, facilitating comprehensive study of single- and multi-factorial relationships between arterial system properties and cardiac structure and function. In the present study we introduce and demonstrate the CircAdapt-TL model (Fig 1): a whole-circulation model with an integrated segmental transmission line (TL) module, describing vascular wave propagation, reflection and transmission.

We verify the numerical implementation of the TL model by a benchmark comparison of the model to the established pulse wave propagation (PWP) model of Kroon et al. [11]. Additionally, we demonstrate operation and output of the integrated CircAdapt-TL model, by simulating systemic normotensive- and hypertensive conditions. We evaluate the implications of modelling vascular wave transmission on aortic haemodynamics by comparing simulated left ventricular- and aortic pressure tracings of the integrated CircAdapt-TL model with the tracings obtained with the systemic circulation lumped into the existing CircAdapt non-linear three-element windkessel (3WK) model (i.e., neglecting wave transmission effects). Further evaluation includes WIA applied to simulated carotid arterial pressure and flow waveforms in semi-quantitative comparison with WIA applied to patient measurements.

## Models

### Overview of complete model

Our vascular module describing wave transmission in vascular networks will be integrated into the existing CircAdapt platform (www.circadapt.org). This model platform has a modular setup, currently consisting of a 0D whole-heart mechanics model, valve haemodynamics model, and non-linear three-element windkessel models of the pulmonary and peripheral circulations (Fig 1). In the next section, we introduce the governing equations, modelling assumptions and implementation of our new vascular module in detail.

### New vascular TL–module

To model pressure-flow waves within segments of blood vessels, we assume 1) blood vessels to be thick-walled, longitudinally constrained non-linear elastic tubes, 2) blood to be incompressible and Newtonian and 3) that gravity forces can be neglected. Furthermore, we assume 4) no leakage of blood to small side-branches that are not explicitly modelled. Application of the laws of balance of mass and momentum, and subsequent integration over the tube’s cross-sectional area yield the governing equations [12]:

$$\begin{array}{c} C\frac{\partial p}{\partial t}+\frac{\partial q}{\partial z}=0\;, \end{array}$$ (1)

$$\begin{array}{c} L(\frac{\partial q}{\partial t}+\frac{\partial }{\partial z}\int_{A}v_{z}^{2}dA)+\frac{\partial p}{\partial z}=f\;, \end{array}$$ (2)

where *p* = *p*(*z*, *t*) is the pressure at the axial vessel coordinate *z*, and *q* = *q*(*z*, *t*) the flow rate at that coordinate. Furthermore, *A* denotes cross-sectional lumen area, and *L* and *C* are the tube inertance and compliance per unit length, respectively. Term $L\frac{\partial }{\partial z}\int_{A}v_{z}^{2}dA$ represents the convective acceleration term, with *v*_*z*_ the axial blood velocity. Term *f* represents friction force per unit volume caused by viscous properties of the blood, defined *f* = 2*πr*_0_*τ*_w_/*A*_0_ [13]. Here, symbol *τ*_w_ denotes wall shear stress, *r*_0_ reference radius, and *A*_0_ reference cross-sectional lumen area, respectively. After neglecting the convective acceleration term and assuming an approximate velocity profile to estimate *τ*_w_ [13], the governing equations can be rewritten to the telegrapher’s equations:

$$\begin{array}{cc} & -\frac{\partial q}{\partial z}=C\frac{\partial p}{\partial t}\;, \end{array}$$ (3)

$$\begin{array}{cc} & -\frac{\partial p}{\partial z}=L\left(\alpha_{0}\right)\frac{\partial q}{\partial t}+R\left(\alpha_{0}\right)q, \end{array}$$ (4)

with *L*(*α*_0_) and *R*(*α*_0_) a characteristic Womersley number-dependent inertance and resistance term, defined by

$$\begin{array}{cc} L(\alpha_{0}) & =g\left(\alpha_{0}\right)\;\frac{\rho }{A_{0}}\;, \end{array}$$ (5)

$$\begin{array}{cc} R(\alpha_{0}) & =h\left(\alpha_{0}\right)\;\frac{8\pi \eta }{A_{0}^{2}}\;,\text{with} \end{array}$$ (6)

$$\begin{array}{cc} \alpha_{0} & =r_{0}\sqrt{\rho \omega_{0}/\eta }\;. \end{array}$$ (7)

The functions *g*(*α*_0_) and *h*(*α*_0_) were derived by Bessems et al. [13] and are detailed in S1 Text, Section ‘Derivation of the attenuation constant, wave speed and wave impedance’. The characteristic Womersley number (*α*_0_) describes the ratio of instationary inertia forces and viscous forces, governed by vessel radius (*r*_0_), characteristic angular frequency (*ω*_0_ = 2*π*/*T*, with *T* the cardiac cycle duration), blood dynamic viscosity (*η*) and blood density (*ρ*), respectively (Table 1).

**Table 1:** Parameters relevant for the transmission line model.

Symbol	Value or expression	Unit	Meaning	Reference
*A*_0_, *r*_0_	See Table B, C in S1 Text	m^2^ or m	Reference lumen area or vessel radius	[14, 15]
*α*_0_	$\alpha_{0}=r_{0}\sqrt{\rho \omega_{0}/\eta }$	-	Characteristic Womersley number	[13]
*b*	0.02	-	Collapsible tube fraction	Assumed
Δ*t*	0.001	s	Time step	Assumed
Δ*z*	0.02	m	Element size	Assumed
*l*_AV_	$6q_{\text{AV}}^{1/3}$	m	Characteristic vessel bed length	Assumed
*k*	See Table B, C in S1 Text	-	Vessel stiffness coefficient	[16], Assumed
*l*	See Table B, C in S1 Text	m	Vessel length	[14, 15]
*η*	0.003	Pa s	Blood dynamic viscosity	[17]
*p*_0,a_	105 (REF), 135 (HYP)	mmHg	Reference pressure for arteries	Assumed
*p*_0,v_	1.1 (REF), 1.1 (HYP)	mmHg	Reference pressure for veins	Assumed
*q*_AV_	See Table B in S1 Text	ml s^–1^	Mean flow through terminal tube	[18, 19], Assumed
*q*_0_	85	ml s^–1^	Mean systemic flow	[2]
*ρ*	1050	kg m^–3^	Blood density	[17]
*T*	0.85	s	Cardiac cycle duration	[2]
*ω*_0_	7.39	rad s^–1^	Characteristic angular frequency	[13]

REF: Reference simulation. HYP: Hypertension simulation.

To solve the governing equations, we also need a constitutive law to relate (changes in) transmural pressure (*p*_trans_) to (changes in) current cross-sectional area (*A*). The rationale of this method is to calculate *R* and *L* based on an approximated velocity profile for which the viscous boundary layer thickness is approximated for the characteristic frequency [13]. We formulated a non-linear power-law to phenomenologically capture the experimentally observed non-linear pressure-area relation of arteries and veins [16, 20]:

$$\begin{array}{c} \begin{array}{cc} p_{\text{trans}}\left(A\right) & =-p_{\text{ext}}+p_{0}(\left(1+b\right){\left(\frac{A}{A_{0}}\right)}^{1+\frac{k/3-2}{1+b}}-\frac{bA_{0}}{A})\;,\\ & \text{and}\;\\ C & =\frac{dA}{dp_{\text{trans}}}\;, \end{array} \end{array}$$ (8)

with *p*_0_ a reference pressure, *A*_0_ a reference cross-sectional area, and *k* the vessel stiffness coefficient. Furthermore, *b* is a small fraction to simulate collapse of the tube with negative transmural pressure (Table 1) and *p*_ext_ represents a prescribed external pressure (if present). Now we can solve the resulting governing equations in either the time domain or frequency domain [21]. We explicitly chose a time-domain approach, since this permits using non-linear boundary conditions as already present in the CircAdapt platform [2]. Our solving method uses a TL model. A detailed overview of our solving method is provided in S1 Text, Section ‘Solving strategy’.

### Arterio-venous impedance module

The terminal end of a tube was coupled to a non-linear three-element windkessel (3WK) element [10]. We assumed the windkessel compliance to be pressure-dependent, and scaled by an estimate of the tissues’ vessel bed length [22]. As a consequence, wave impedance also becomes pressure-dependent (Fig 1):

$$\begin{array}{c} C_{\text{AV}}=l_{\text{AV}}\frac{dA}{dp_{\text{AV}}}\;\text{and}\;Z_{\text{wave},\text{AV}}=\sqrt{\frac{\rho }{A}\frac{dp_{\text{AV}}}{dA}}\;, \end{array}$$ (9)

with AV, the subscript for the arterial and venous contributions, i.e. AV = [art, ven]. Such approach enables simulating large changes in haemodynamic load (e.g. exercise or hypertension) without requiring to manually adjust the 3WK parameter values. The derivatives in the aforementioned equations were calculated at the connection point (i.e. a node) of a tube with a 3WK, using the constitutive relation as given in Eq 8. The parameter *l*_AV_ represents the characteristic length of a peripheral bed. We estimated the vessel bed length using the relation given by $l_{\text{AV}}=6q_{\text{AV}}^{1/3}$, with *q*_AV_ the mean peripheral flow through any terminal tube. To obtain first-order approximations of *l*_AV_ among all peripheral beds, we utilised this relation in conjunction with flow distribution estimates as reported in Table B in S1 Text. Furthermore, using a physiology textbook [18], we estimated that in rest 21% of the cardiac output is directed to the head, 47% to the abdomen, 18% to the pelvis and lower extremities, and 14% to the upper extremities, respectively. The peripheral resistance (*R*_p_) was defined via a flow source, controlled by the instantaneous arterio-venous pressure difference (Fig 1) [10]:

$$\begin{array}{c} R_{p}=\frac{p_{\text{art}}-p_{\text{ven}}}{q_{\text{AV}}}\;. \end{array}$$ (10)

### Cardiac module

The atria and ventricles of the heart were modelled as contractile chambers. The ventricles are surrounded by three cardiac walls: the left ventricle free wall, interventricular septum and right ventricle free wall (Fig 1). Ventricles are mechanically coupled, based on force equilibrium in the junction of the ventricular walls [9]. The atria are surrounded by the left atrial wall and right atrial wall (Fig 1). The cardiac chambers are considered as contractile cavities formed by the one-fibre model, relating myofibre stress to cavity pressure using the assumption that myofibre stress is homogeneously distributed within the myocardial wall [23]. The phenomenological model of myofibre mechanics was previously described [2]. The one-fibre model is used to calculate myofibre stress from myofibre strain. Total Cauchy myofibre stress experienced by cardiac tissue comprises of a summation of active stress, present in the actin filaments and separate microstructural contributions (i.e. titin and the extracellular matrix, assumed to act in parallel). Transmural pressure is calculated from wall tension, derived from total Cauchy stress and wall curvature using Laplace’s law [2]. Cavity pressures are calculated by adding the transmural pressures to the pericardial pressure surrounding the myocardial walls. As commonly used in other cardiac models, the pericardium was assumed a compliant bag, modelled using a non-linear relation relating pericardial pressure and volume [24]. The pulmonary circulation was modelled as 3WK (see Section ‘Arterio-venous impedance module’), connecting the pulmonary artery with the pulmonary veins [25]. Full details of the cardiac model can be retrieved from Walmsley et al. [2] and Lumens et al. [9].

### Valve module

Valve flow (*q*_valve_) was generated using the unsteady Bernoulli equation, assuming incompressibility and inviscid, irrotational flow:

$$\begin{array}{c} \begin{array}{cc} \frac{\rho l_{\text{valve}}}{A_{\text{valve}}}\frac{\partial q_{\text{valve}}}{\partial t} & =\Delta p-\frac{1}{2}\rho \;q_{\text{valve}}\left|q_{\text{valve}}\right|(\frac{1}{A_{\text{valve}}^{2}}-\frac{1}{A_{p}^{2}})\;, \end{array} \end{array}$$ (11)

with the term on the left hand side the unsteady inertia, governed by blood density, effective valve length (*l*_valve_) and valve cross-sectional area (*A*_valve_) [1]. The first term on the right hand side denotes the pressure difference (Δ*p*) and the second term is the change in kinetic energy, with *A*_valve_ and *A*_p_ cross-sectional areas of the valve and proximal to the valve, respectively. For *A*_valve_, a phenomenological valve opening and closing function depending on the pressure gradient was used [1]. In case Δ*p* > 0, *A*_valve_ instantaneously increases towards an effective valve area representing a completely opened valve. In case Δ*p* < 0, on the other hand, flow gradually decreases due to inertia. Furthermore, *A*_valve_ gradually decreases towards a quasi-closed state, with small leakage to avoid division by zero [1].

### Simulations and analysis

#### Benchmark comparison

First, we evaluated the TL model’s numerical framework in a benchmark comparison, comparing arterial pressure and flow waveforms generated by on the one hand the TL model and those generated by the PWP model, developed by Kroon et al. [11]. The numerical framework of this PWP model was previously validated in Boileau et al. [26] against *in vitro* experiments, whereas Bessems et al. [13] confirmed the validity of the approximate velocity profile against Womersley theory. In the benchmark comparison, we considered large central arteries (vessel diameters between 15 and 30 mm) as well as smaller arteries of the left arm (vessel diameters between 2 and 11 mm, Table B in S1 Text). Furthermore, the same set of equations (i.e. the balance equations and the constitutive law) was solved for the PWP- as well as for the TL model. We also kept boundary conditions (i.e. constitutive law and 3WK parameters) equal between the PWP- and TL model. At the proximal aorta we prescribed a periodical flow wave (*q*_inflow_) with a period of 0.85 s. This flow wave was composed of a half-sinusoidal wave with a duration (*t*_c_) of 0.3 s and a peak flow rate (*q*_p_) of 350 ml s^–1^, whereas flow was zero in the rest of the period,

$$\begin{array}{c} q_{\text{inflow}}\left(t\right)=\{\begin{array}{cc} q_{p}\sin(\frac{\pi t}{t_{c}}) & \text{if}\;0\leq t\leq t_{c}\\ 0 & \text{if}\;t>t_{c}\;. \end{array} \end{array}$$ (12)

The PWP model used a simplified trapezoidal scheme for spatial discretisation and a second-order backward difference scheme for time discretisation. For each tube in the domain, agreement between models in terms of pressure and flow, was quantified by calculation of root mean square errors (*ϵ*) and relative errors (*δ*):

$$\begin{array}{cc} \epsilon_{p,n} & =\sqrt{\frac{\sum_{t=0}^{T}{\left(p_{\text{TL}}\left(t\right)-p_{\text{PWP}}\left(t\right)\right)}^{2}}{N_{t}}}\;,\\ \epsilon_{q,n} & =\sqrt{\frac{\sum_{t=0}^{T}{\left(q_{\text{TL}}\left(t\right)-q_{\text{PWP}}\left(t\right)\right)}^{2}}{N_{t}}}\;,\\ \delta_{p,n} & =\frac{1}{N_{t}}\sum_{t=0}^{T}|\frac{p_{\text{TL}}\left(t\right)-p_{\text{PWP}}\left(t\right)}{p_{\text{PWP}}\left(t\right)}|\cdot 100\%\;,\text{and}\\ \delta_{q,n} & =\frac{1}{N_{t}}\sum_{t=0}^{T}|\frac{q_{\text{TL}}\left(t\right)-q_{\text{PWP}}\left(t\right)}{{\text{max}}_{n}(q_{\text{PWP}}\left(t\right))}|\cdot 100\%\;, \end{array}$$ (13)

with *n* the tube reference number, *T* the cardiac cycle duration and *N*_*t*_ the number of time points for which a comparison is made. A complete overview of the benchmark comparison setup is provided in S1 Text, Section ‘Benchmark comparison between TL and PWP model’.

#### Simulation of arterial and venous haemodynamics using the CircAdapt–TL model

**Fig 2:**
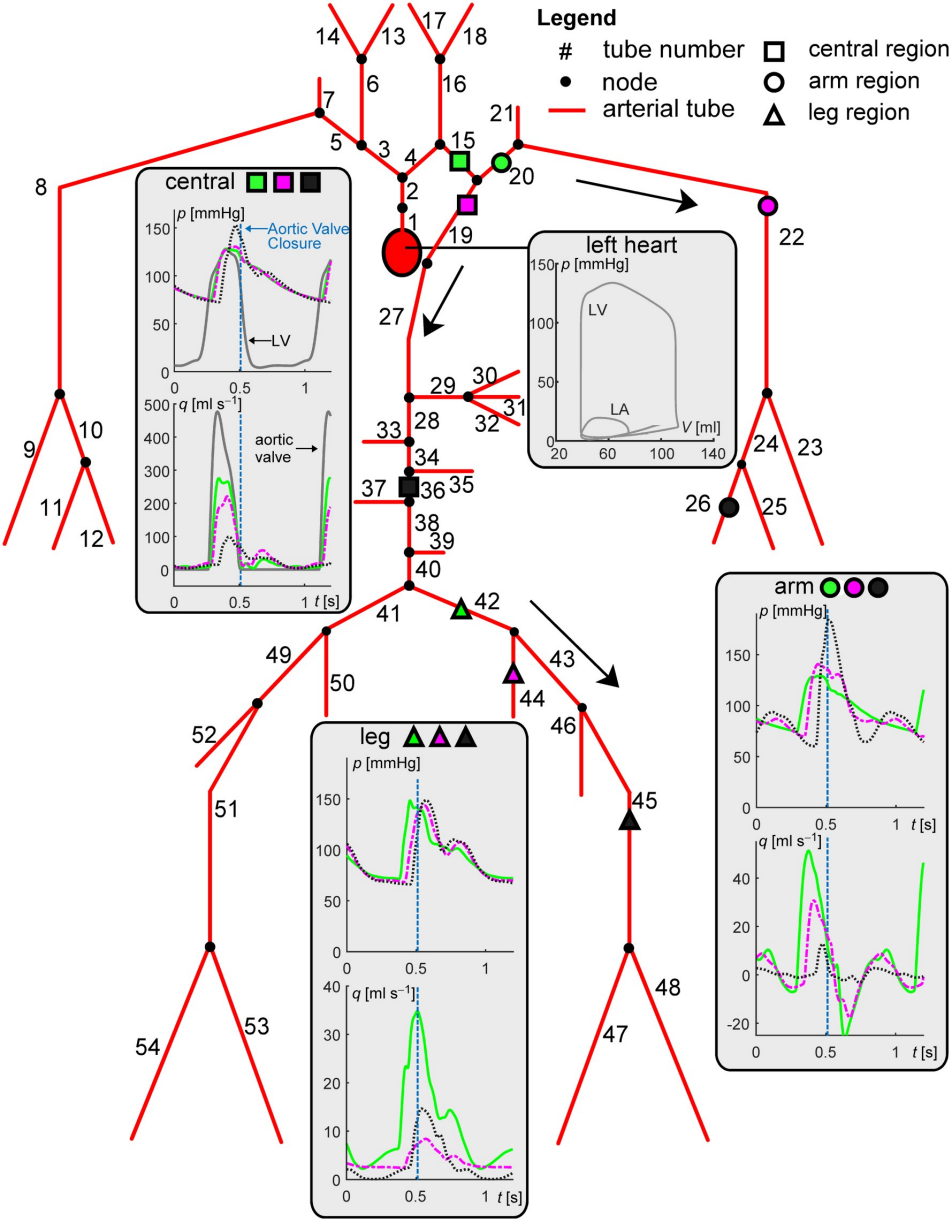
Overview of the complete arterial model domain. Geometrical and mechanical properties of modelled arteries are given in Table B in S1 Text. Panels display pressure and flow waveforms for three regions (i.e. central, arm and leg, indicated using symbol and colour coding). Grey curves display left ventricular (LV) pressures and volumes, left atrial (LA) pressures and volumes as well as aortic valve flow. Line type indicates the distance from the heart within a region; proximal: solid line, intermediate: dash-dotted line, distal: dotted line, respectively. Arrows indicate the direction of mean blood flow. Aortic valve closure is indicated by the vertical blue dashed lines.

**Fig 3:**
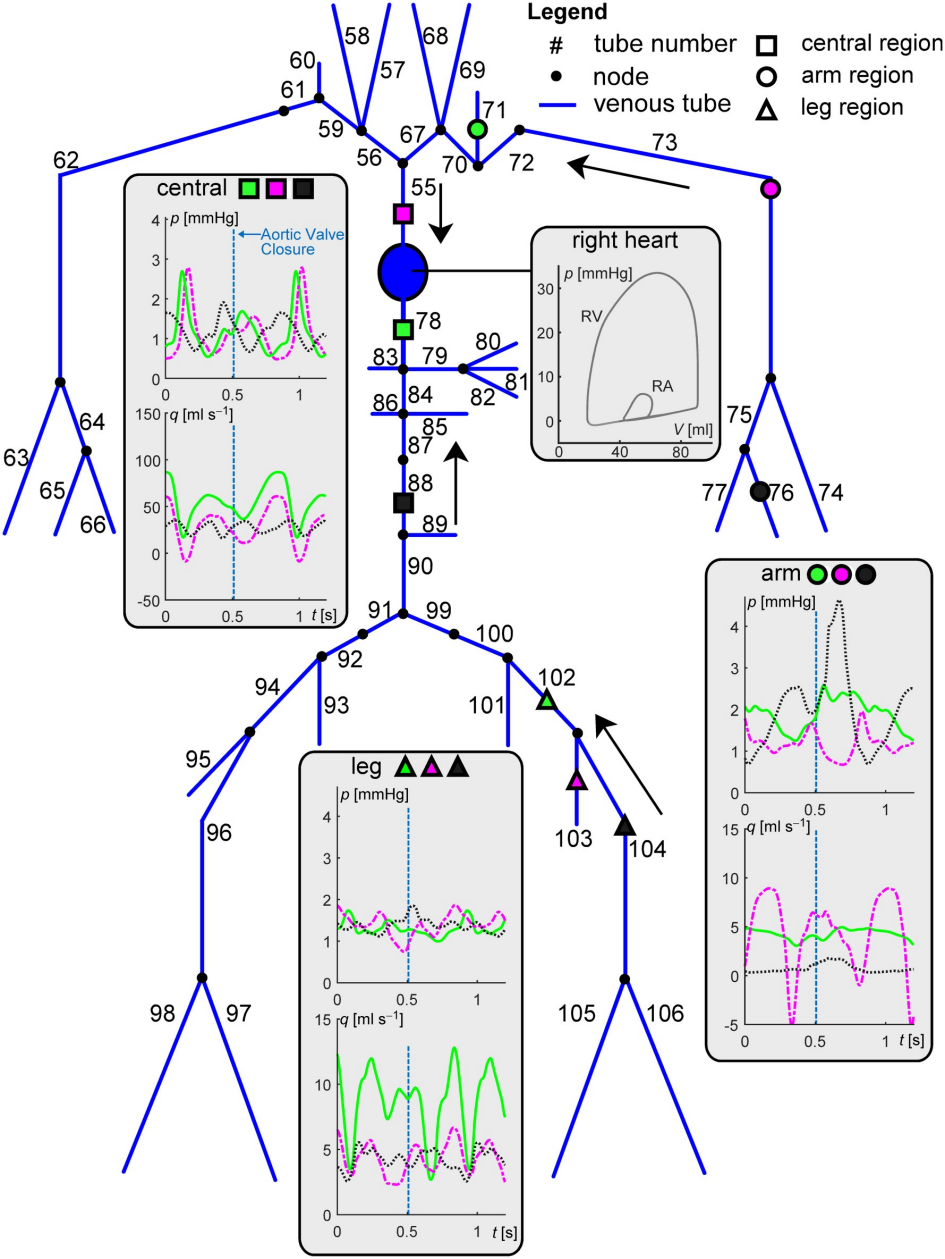
Overview of the complete venous model domain. Geometrical and mechanical properties of modelled veins are given in Table C in S1 Text. Panels display pressure and flow waveforms for three regions; central, arm and leg, indicated using symbol and colour coding. Line type indicates the distance from the heart within a region; proximal: solid line, intermediate: dash-dotted line, distal: dotted line, respectively. Grey curves display right ventricular (RV) pressures and volumes, right atrial (RA) pressures and volumes. Arrows indicate the direction of mean blood flow. Aortic valve closure is indicated by the vertical blue dashed lines.

We first show the applicability of the CircAdapt–TL model in terms of simulation of pressure and flow waveforms throughout arteries and veins as well as changes in wave dynamics when simulating systemic hypertension. Furthermore, we compare LV and aortic pressure tracings of the CircAdapt–TL model with those obtained when lumping the systemic arterial and venous blood vessels into a single 3WK. For the first purpose, we implemented an arterial tree (Fig 2 and Table B in S1 Text), for which the morphological and mechanical properties were based on the work of Reymond et al. [15], and a venous tree (Fig 3 and Table C in S1 Text), based on the work of Müller and Toro [14]. Since in the present study we were not interested in simulating pressure and flow in cerebral and coronary vessels, we excluded these vessels from our model domain. Vessel stiffness coefficients (*k*) for the aortic segments as well as arteries of the left and right arm were as used in the benchmark comparison. Furthermore, for the arteries of the leg (i.e. the iliac, femoral and tibial arteries), we chose *k* equal to 30, based on data of Hayashi et al. [16]. Given the lack of human data on veins, a fixed *k*-value of 10 was chosen for all veins in the model domain.

We performed the following sets of simulations.

1. Using the CircAdapt–TL model, we performed:
  - A reference simulation (REF–TL) in which we model the case of normal arterial stiffness. For this simulation, we kept reference pressure (*p*_0_) at 105 mmHg and vessel stiffness coefficient (*k*) as given in Tables A and B in S1 Text. According to clinical standards, we characterised arterial stiffness by calculating the carotid-femoral pulse wave velocity (PWV). We obtained PWV by dividing the fixed path length between the terminal nodes of the tubes that modelled the carotid artery and femoral artery by the pulse transit time between these nodes. The pulse transit time was obtained as the foot-to-foot time difference between the respective pressure waveforms. Foot detection was performed using the maximum of the 2^nd^-order derivative of the pressure waveform.
  - A systemic hypertension simulation (HYP–TL), in which we model a hypertensive situation. For this simulation, we increased *p*_0_. Such increase in *p*_0_ can physiologically be interpreted as an increase in peripheral resistance. Furthermore, vascular stiffness parameter *k* of all systemic arterial segments was increased by a factor Δ*k*. The latter increases the non-linearity of the pressure-area relation (Eq 8), and models an increase in material stiffness of the vessel wall. For the HYP simulation, we imposed [*p*_0_, Δ*k*] = [135 mmHg, + 6]. This resulted in a blood pressure exceeding 140/90 mmHg, a situation defined as hypertension according to the European Society of Hypertension/European Society of Cardiology guidelines [27].
2. Using the CircAdapt–3WK model, we performed
  - A reference and hypertension simulation. Now, the systemic circulation was lumped into a 3WK (REF–3WK and HYP–3WK, see S1 Text, Section ‘Simulations performed using the CircAdapt–3WK model’).

For the REF–TL and HYP–TL simulations, we compared pressure and flow waveforms for cardiac chambers, central arteries and veins, as well as trans-valvular flows. Furthermore, haemodynamic indices (i.e. diastolic, systolic blood pressures, pulse wave velocity) were compared between the REF–TL and HYP–TL simulation. Systolic and diastolic aortic blood pressures were calculated from the tube that mimics the ascending aorta (i.e. tube #1, Fig 2). The following analyses were conducted to assess the implications of using the TL model on aortic haemodynamics. First, we compared the morphology of pressure tracings of the LV and aorta obtained using the CircAdapt–TL model with those from the CircAdapt–3WK model. Second, wave intensity analysis was performed in the common carotid artery. Wave intensity analysis provides an approach to determine the characteristics of the primary wave originating from wave reflection, (i.e. termed the backward compression wave (BCW)) [7]. Wave intensity may be defined as the rate of energy transfer per unit area, often given in units of [W m^–2^ s^–2^] [28]. Wave intensity is positive (d*I*^+^) for a forward running wave, and negative (d*I*^−^) for a backward running wave. Furthermore, net wave intensity (d*I*) is defined as the sum of backward and forward wave intensity, respectively. Using the derived wave intensity tracings, we qualitatively assessed changes in arrival time and intensity of the BCW between the normotensive- and hypertensive situations. Finally, we compared between simulated wave intensity tracings and those obtained in patients. Details regarding derivation of d*I*^+^, d*I*^−^, and d*I* are provided in S1 Text, Section ‘Calculation of wave intensity’.

#### Numerical implementation

All simulations were performed in MATLAB 2015a (The MathWorks, Natick, MA, USA) on a standard personal computer running an Intel^®^ Core i7™processor and 8.00 GB RAM. Blood viscosity was kept at 3 ⋅ 10^−3^ Pa s, blood density was kept at 1050 kg m^–3^, and collapsible tube fraction *b* was kept at 0.02 (Table 1). We chose a time step (Δ*t*) of 1 ms. Furthermore, for the simulations performed using the CircAdapt–TL model, we chose an element size (i.e. Δ*z*, indicating the distance between nodes) of 0.02 m. This value was chosen as a trade-off between on the one hand being able to capture geometric tapering of blood vessels, and on the other hand to restrain simulation time. For both the benchmark evaluation and the simulations that were performed using the CircAdapt-TL and CircAdapt-3WK models, we ensured that numerical convergence was achieved (i.e. negligible change in pressure and flow waveforms when further decreasing Δ*t* and Δ*z*).

## Results

### Benchmark comparison

**Fig 4:**
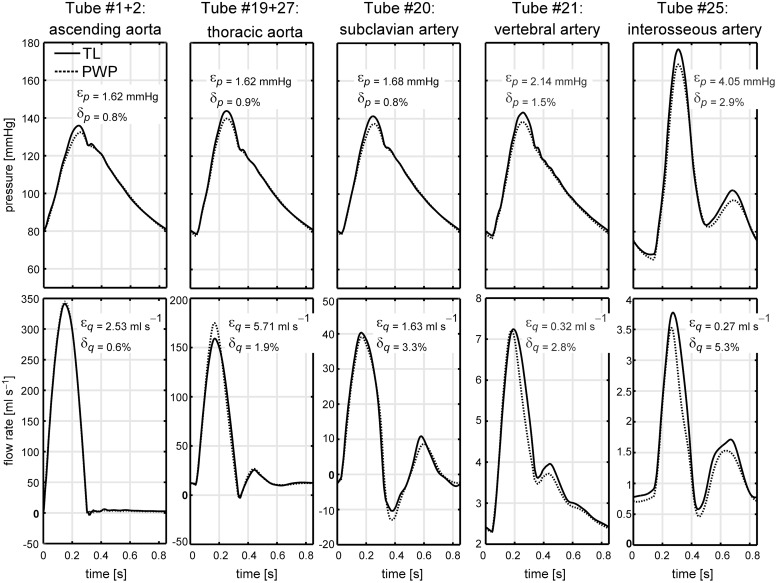
Results of the benchmark comparison. Pressure (*p*) and flow (*q*) waveforms generated by the transmission line (TL) model and 1D pulse wave propagation model (PWP) model are shown for various locations along the arterial domain. Agreement between *p* and *q* waveforms is expressed by root mean square errors *ϵ*_*p*_ and *ϵ*_*q*_, as well as relative errors *δ*_*p*_ and *δ*_*q*_.

Agreement between pressure and flow waves of the TL model and PWP model for five tubes in the model domain is graphically depicted in Fig 4. Root mean square errors (Eq 13) for pressures and flows for all tubes are given in Table 2.

**Table 2:** Benchmark comparison between pressure and flow waveforms of the new transmission line (TL) model and established pulse wave propagation (PWP) model.

tube #	1+2	4	15	19+27	3	16	20	21	22	23	24	26	25
*ϵ*_*p*_ [mmHg]	1.62	1.65	1.68	1.62	2.36	1.68	1.68	2.14	2.96	3.96	3.68	4.03	4.05
*ϵ*_*q*_ [ml s^–1^]	2.53	21.20	4.41	5.71	3.02	0.87	1.63	0.32	0.92	0.22	0.48	0.30	0.27
*δ*_*p*_ [%]	0.8	0.8	0.8	0.9	1.2	0.8	0.8	1.5	1.5	2.7	2.2	2.8	2.9
*δ*_*q*_ [%]	0.6	5.6	1.1	1.9	4.5	2.2	3.3	2.8	3.5	3.2	3.1	3.7	5.3

Root mean square errors for pressure (*ϵ*_*p*_) and for flow (*ϵ*_*q*_), as well as their relative errors (*δ*_*p*_ and *δ*_*q*_) describe agreement between both models. Agreement was calculated for the element located at the center of the tube.

Between models, we found good agreement in terms of pressure and flow waveforms for proximal arteries (e.g. aorta, carotid, subclavian and vertebral arteries), expressed by relative errors *δ*_*p*_ ≤ 1.5% and *δ*_*q*_ ≤ 5.6% At the distally located interosseous artery, the difference between both models slightly increased, expressed by *δ*_*p*,25_ equal to 2.9% and *δ*_*q*,25_ equal to 5.3%. Nevertheless, the shape of the pressure and flow waveforms as well as absolute systolic and diastolic pressure and flow values were highly similar (Fig 4).

### Simulation of normotension and hypertension

In Figs 2 and 3, pressure and flow waveforms in normotension are displayed for arteries and veins at three regions (i.e. the central-, arm- and leg region). Arterial pressure waveforms at distal locations are characterised by an increase in pressure amplitude, as well as a reduction in peak width. The arterial pressure waveforms at distal locations show a more prominent dicrotic notch compared to the pressure waves at proximal locations. For veins, a biphasic pressure waveform can be distinguished, with venous flow and pressure in anti-phase (Fig 3).

In the REF–TL simulation, pulse wave velocity (PWV) was 5.5 m s^–1^, representing a PWV value commonly found in subjects aged < 30 years [29]. For the HYP simulation, pulse wave velocity (PWV) was 8.0 m s^–1^, representing a PWV value clinically associated with early aortic stiffening, and commonly found in subjects aged > 50 years [29].

**Fig 5:**
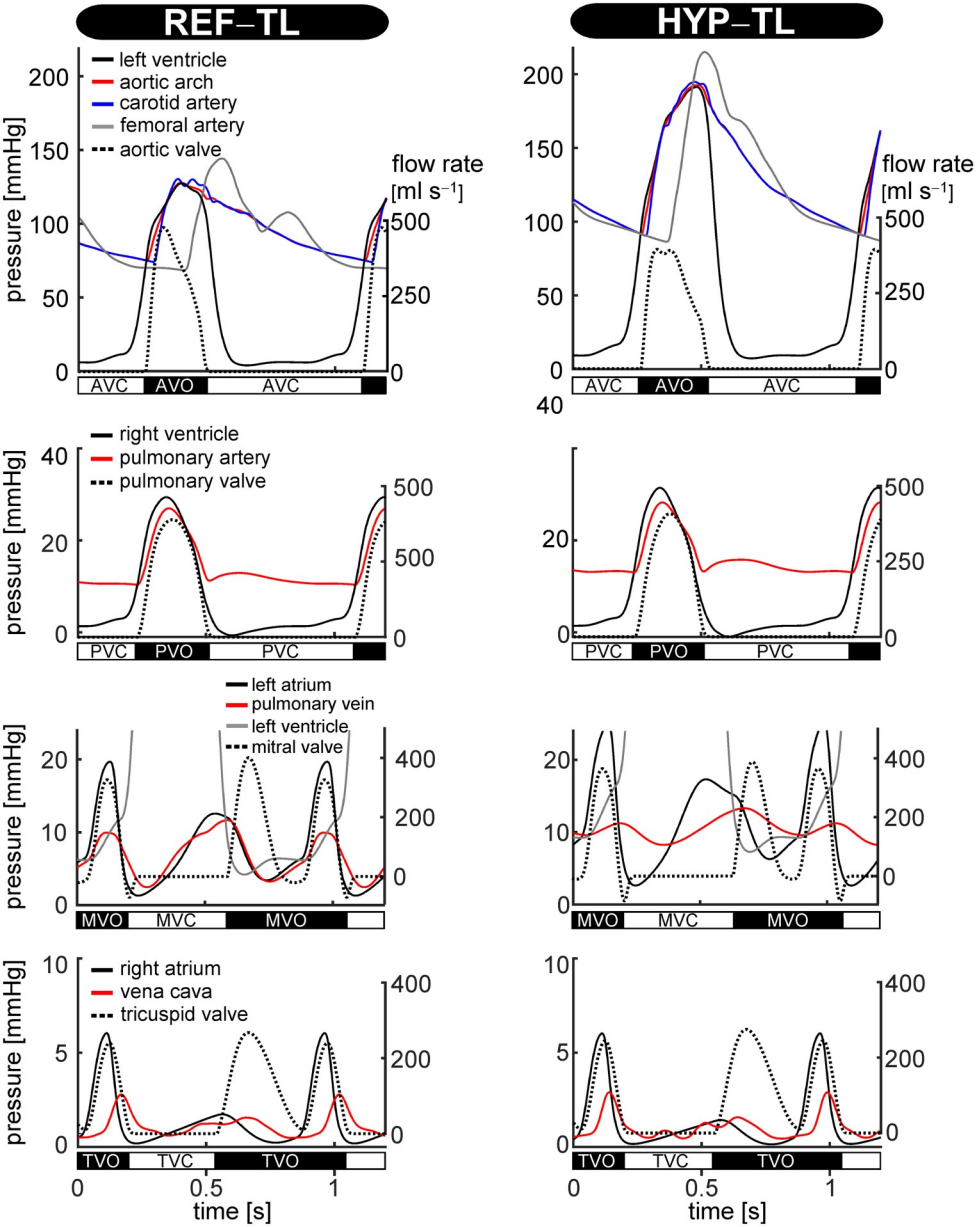
CircAdapt–TL model simulated time courses of pressure and flow for the reference (REF–TL) simulation (left), and the hypertension (HYP–TL) simulation (right). Curves display time courses of pressure (solid lines) and flow (dotted lines) of the left ventricle and large arteries; the right ventricle and pulmonary artery; the left atrium and pulmonary vein; and the right atrium and vena cava, respectively. Valve states (i.e. open or closed) are indicated. These have been determined exactly from the valve model orifice area data, not from the pressure or flow signals. AVC: aortic valve closed, AVO: aortic valve open, MVC: mitral valve closed, MVO: mitral valve open, PVC: pulmonary valve closed, PVO: pulmonary valve open, TVC: tricuspid valve closed, TVO: tricuspid valve open.

The blood pressure values in the REF–TL simulation were within the normal range (Table 3). As shown in Fig 5, simulating systemic hypertension (HYP) caused arterial pressure to increase. This resulted in an increase in left ventricular pressure and left atrial pressure, whereas pulmonary artery pressure and pulmonary venous pressure slightly increased (Fig 5). The HYP–TL simulation showed an increase in systolic blood pressure (*p*_sys_) from 128 to 193 mmHg and an increased diastolic blood pressure (*p*_dia_) from 75 to 92 mmHg (Table 3).

**Table 3:** Haemodynamic indices in the reference (REF) and hypertensive (HYP) simulations.

Index	REF	HYP	Unit	Meaning
*p*_sys_	128	193	mmHg	Systolic pressure in the aorta
*p*_dia_	75	92	mmHg	Diastolic pressure in the aorta
*p*_pulse_	53	101	mmHg	Pulse pressure in the aorta
PWV	5.5	8.0	m s^–1^	Carotid–femoral pulse wave velocity

**Fig 6:**
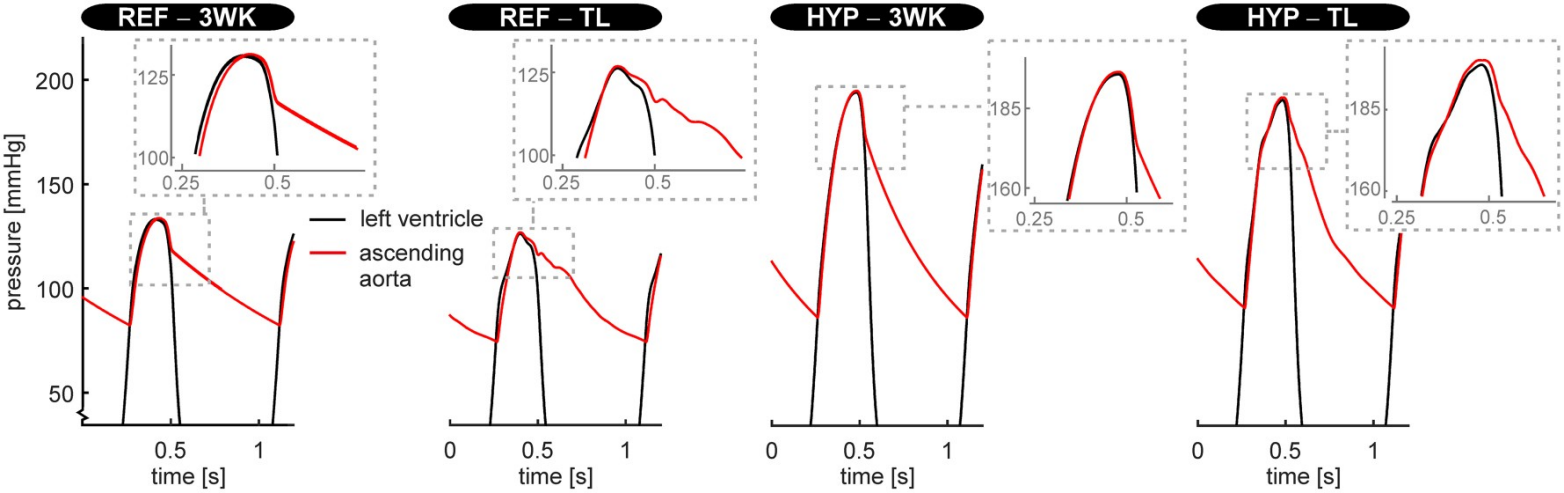
Left ventricular- and aortic blood pressure tracings simulated using, on the one hand, the CircAdapt model with the systemic circulation lumped into a non-linear three-element windkessel model (3WK) and, on the other hand, CircAdapt with the systemic circulation represented by transmission lines (TL). Pressure tracings are given for the normotensive (REF–3WK and REF–TL, respectively) and hypertensive situation (HYP–3WK and HYP–TL, respectively).

Fig 6 shows LV and ascending aortic pressure tracings obtained using the CircAdapt–3WK model and the CircAdapt–TL model, respectively. The aortic pressure tracings of the REF–TL and HYP–TL simulation showed pressure augmentation (i.e. a systolic pressure boost) as well as an dicrotic notch, whereas for CircAdapt–3WK model simulations, these waveform characteristics were absent. In the REF–TL simulation, systolic pressure augmentation occurred after time of peak systolic pressure, whereas in the HYP–TL simulation this occurred prior to the time of peak systolic pressure.

**Fig 7:**
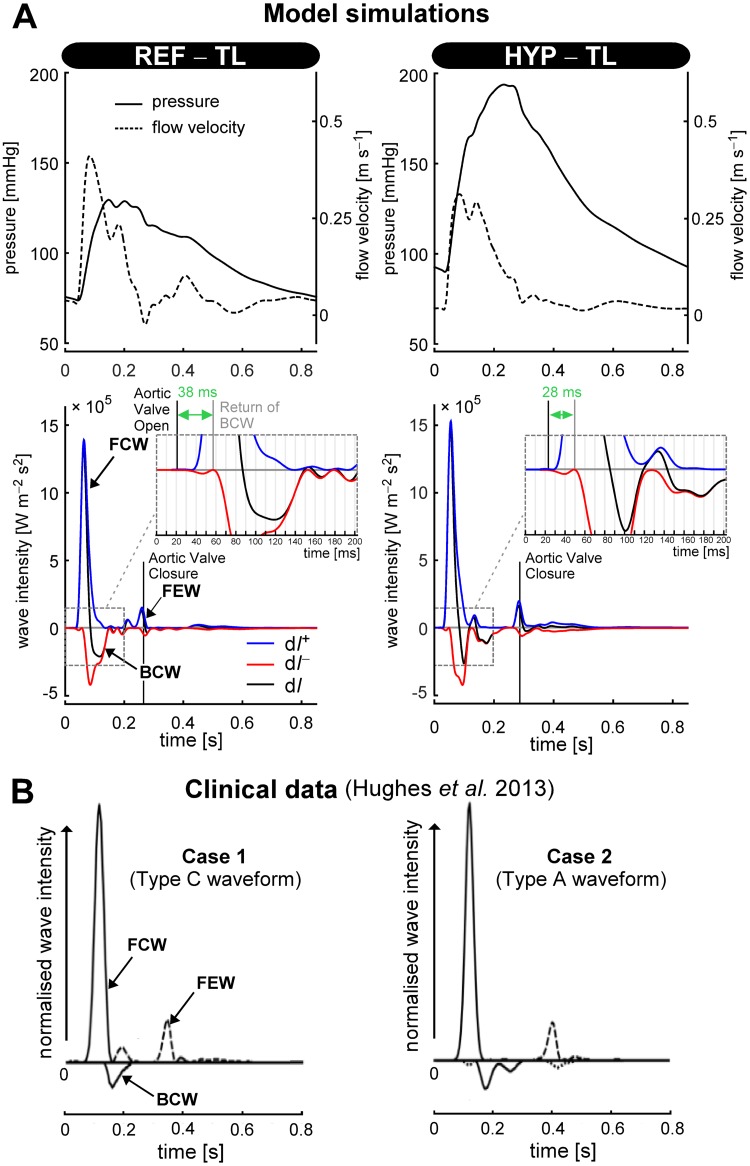
Wave intensity analysis. **A**: Carotid arterial pressure- and flow velocity waveforms of the reference (REF–TL) and hypertensive (HYP–TL) simulations, and corresponding wave intensity traces. **B**: Normalised carotid arterial wave intensity traces of two patients (adapted from [31], with reference to type C- and A-waveforms). Abbreviations: d*I*^+^, d*I*^−^ and d*I* denote forward-, backward-, and net wave intensity, respectively. FCW, BCW, FEW: forward compression wave, backward compression wave, and forward expansion wave, respectively.

Wave intensity tracings (d*I*^+^, d*I*^−^, and d*I*, respectively) of the REF–TL and HYP–TL simulation were calculated for the left common carotid artery (Fig 7A). The carotid arterial wave intensity tracings of the REF–TL simulation indicate a forward compression wave (FCW) followed by a backward compression wave (BCW). At end-systole, there is a forward expansion wave (FEW) associated with the deceleration of the rate of myocardial contraction [30]. In the REF–TL simulation, the onset of the BCW occurred 38 ms after onset of left ventricular ejection, whereas for the HYP–TL simulation the delay was 28 ms (Fig 7A). Peak wave intensity of the BCW was approximately equal for the HYP–TL simulation (4.19 ⋅ 10^5^ W m^–2^ s^–2^) as compared to the REF–TL simulation (4.23 ⋅ 10^5^ W m^–2^ s^–2^) (Fig 7A). Overall, the pattern of the simulated wave intensity tracings was similar to measured carotid arterial wave intensity tracings as reported by Hughes et al. [31] (Fig 7B).

## Discussion

We integrated a transmission line (TL) model into the existing CircAdapt platform of whole-heart mechanics. The resulting benchmarked CircAdapt–TL model now also describes vascular wave transmission. The presented model and simulation/validation results bring the following innovative steps forward. First, our model platform allows for computationally efficient simulation of cardiac mechanics and vascular haemodynamics. CircAdapt–TL, as implemented in MATLAB, simulates a single cardiac cycle in 6 s, whereas the CircAdapt-3WK model requires 4 s but does not model wave transmission. We consider our TL implementation of a distributed model well-justified based on the acceptable increase in computational time. Second, the modular structure of CircAdapt–TL renders it easy to change the model domain (e.g. through user-defined assembly of network connections of the various modules). This versatility comes at hand when handling the model in a non-engineering environment, for example in a classroom of cardiologists in training. Third, the coupling of the extension of CircAdapt heart [9] with our framework to capture large vessels, in conjunction with modest increase in computational demand, facilitates extensive uncertainty quantification and sensitivity analysis of detailed haemodynamics mechanisms.

The field of distributed modelling of vascular wave transmission considers continuous pulse wave propagation (PWP) models that compute pressure and flow using numerical techniques such as finite differences or the method of characteristics [32], and models that use transmission line (TL) theory based on the telegraph equations [17, 33, 34]. We chose the TL model for its reduced computational cost as compared to models using the method of characteristics, its capability of operating in the time-domain, and its compatibility with the existing CircAdapt model [2, 33].

The validity of our TL model’s numerical implementation was assessed by comparing pressure and flow waveforms generated with the TL model with those generated by the validated model of Kroon et al. [11]. For adequate comparison, an exact same model domain ranging from the large central arteries towards the smaller arteries of the left arm was chosen. Moreover, for both models boundary conditions defining proximal inflow, vessel wall mechanics, and outflow conditions were kept identical. Remaining differences in pressure and flow waveforms between both models may be attributed to the fact that in deriving the propagation constant, our TL model neglects the higher order terms to render the attenuation constant frequency-independent (see S1 Text, Section ‘Derivation of the attenuation constant, wave speed and wave impedance’). This assumption is justified for high frequencies, but may become questionable for low frequencies. For the PWP model of Kroon et al. [11], such assumption was not made, since the attenuation in that model is incorporated by wall shear stress [11].

For the CircAdapt–TL model, pressure and flow waveforms of the arteries showed physiologically realistic behaviour in both time and position along the modelled domain. The morphology of arterial pressure waveforms of the aorta towards the radial and ulnar arteries resemble pressure waveforms measured during pressure catheter withdrawal from the aorta to the radial artery in man [35]. Venous pressure and flow waveform morphologies were similar to the ones obtained in the computational model study of Müller and Toro [14]. We found a similar venous pressure pulsatility (i.e. the maximum–minimum difference in venous pressure) as these investigations reported (i.e. between 1.2 and 2.3 mmHg in the central veins and between 0.9 and 3.9 mmHg in veins of the arm, respectively). In the reference (REF–TL) simulation, systolic and diastolic pressure in the aorta as well as pulse wave velocity were within normal ranges. In the hypertension (HYP–TL) simulation, blood pressure clearly increased indicated by the aortic systolic and diastolic pressures of 193 and 92 mmHg. The pulse pressure increase in the systemic hypertension simulation to 101 mmHg is considered high given the moderate increase to a pulse wave velocity of 8.0 m s^–1^ [29]. In our model, such large increase in pulse pressure could be caused by the fact that we did not incorporate the dilatation of arteries caused by vascular remodelling in hypertension [29, 36].

For the hypertension simulation, wave intensity analysis revealed earlier arrival of a backward compression wave (Fig 7A), consistent with human measurements [37]. The simulated ascending aortic blood pressure waveform changed from a type C waveform in the REF–TL simulation, most often seen in young adults under 30 years of age, to a type A waveform in the HYP–TL simulation, most often seen in subjects aged 40 to 65 years [38]. Although the classification of pressure waveforms according to systolic pressure augmentation may appear subtle, the clinical-epidemiological field assesses indices derived from systolic pressure augmentation for cardiovascular risk stratification [39, 40]. Simulated carotid arterial wave intensity tracings appeared similar to illustrative examples of measured tracings (Fig 7B). In the HYP–TL simulation, a so-called mid-systolic forward expansion wave was present, similar to patient case 1 (around *t* = 0.2 s) as described in [31]. Though this illustrates the level of detail possible in model-based haemodynamic studies, the mechanistic interpretation of e.g. a mid-systolic forward expansion wave is beyond the scope of this method paper.

With lumping the systemic vessels into a 3WK, the aortic pressure tracings lost the typical dynamics around the dicrotic notch, which were present in the REF–TL and HYP–TL simulations. Moreover, the (early) systolic pressure augmentation in the LV and aortic pressure tracings was absent for the pressure tracings of the REF–3WK and HYP–3WK simulation (Fig 6). Given the interest in parameters such as systolic pressure augmentation, we believe that using distributed models like ours, for studies on heart-vessel interaction, may be preferable over using 3WK models. However, future experimental studies and clinical comparisons are needed to appraise the added value of distributed models over 3WK models in testing heart-vessel interaction hypotheses.

### Limitations

A simplification in the TL model is that convective acceleration is neglected. However, the influence of convective acceleration on arterial pressures and flows is believed to be small [41]. Moreover, it was found that inclusion of convective acceleration in an arterial model domain, similar to the one used in the present study, changed pressure and flow waveforms in the various arteries only slightly (i.e. a root mean square error of 1.3 mmHg for thoracic aortic pressure waveform and 11.3 ml s^–1^ for thoracic aortic flow waveform, respectively [15]). We expect, however, that the effect of convective acceleration will be more important when simulating exercise conditions. Therefore, in such studies the modelling error related to convective acceleration needs to be properly considered.

By excluding cerebral and coronary vessels from our model domain, we neglect the presumed influence that wave reflections and re-reflections from head and neck vessels or vessels in the myocardium may have on observed ascending aortic and carotid waveforms [15, 42].

Our model neither contains a skeletal-muscle pump model nor does it incorporate venous valves. Hence, the present vascular model will not account for these functional aspects with postural changes. For studies with emphasis on venous haemodynamics, the CircAdapt platform allows for a straightforward implementation of venous valves, using for instance the existing valve module as a starting point.

Like all distributed models of 1D wave transmission, our model cannot capture the complex pressure losses or local wall shear stresses when applied to disease conditions (e.g. stenosis or aneurysm). This requires either use of calibrated loss models or coupling of detailed 3D models of stenoses and aneurysms to 1D models, respectively [43, 44].

Reymond et al. [45] reported for the case of an apparently healthy aorta, that pressure and flow waveforms from a 3D CFD model and from a 1D PWP model are highly similar. The latter finding supports our notion that, in general, distributed models of wave transmission are well suited to examine and quantify heart-vessel interaction at the level of pressure and flow waveform characteristics.

### Perspectives

The utility of the CircAdapt–TL model should be further tested by direct comparisons against detailed haemodynamic data from humans. We consider the concurrent use of *in vivo* as well as simulated data as most valuable, because both arms bring complementary assets. The model allows error free assessment of phase relationships between signals and *in vivo* data enables characterisation of biological and pathological variability.

In the future, we aim to further extend the CircAdapt–TL model with the cardiac adaptation module of Arts et al. [46]. This module contains a homeostatic control loop that senses offsets in mechanical load (e.g. as present in chronic hypertension), and in response, imposes geometrical (i.e. cavity volume and wall volume) adaptation of the heart. We believe that modelling cardiac adaptation is vital in assessing candidate haemodynamic indices.

Key clinical studies in this field include that of Hashimoto et al. [6]. They found a positive association between left ventricular mass, and wave reflection magnitude derived from pressure and flow velocity measurements, following antihypertensive treatment in left ventricular hypertrophy patients. However, a limitation of such clinical studies is that for non-invasive acquisition, pressure signals are obtained at distal measurement sites (e.g. at the radial artery) and therefore require a transfer function to obtain an estimate of the aortic pressure signal. Moreover, for clinically-gathered data, correct synchronisation of pressure and flow velocity signals is crucial, because only a small (e.g. 5 ms) misalignment can cause substantial changes in derived wave (intensity) quantities [7].

### Conclusions

We validated and incorporated a one-dimensional vascular module into the CircAdapt platform. The resulting CircAdapt–TL model enables fast simulation of whole-heart mechanics, pressure and flow waveforms at various locations along the arterial and venous systems, and allows detailed haemodynamics studies. The CircAdapt–TL model provides a valuable tool for testing hypotheses concerning heart-vessel interaction and evaluating existing haemodynamics indices.

## Supporting information

S1 Text CircAdapt–TL model paper supplement. This document contains a detailed overview of the transmission line (TL) model’s solving strategy, geometrical- and mechanical properties of arterial- and venous segments belonging to the modelled vascular trees, as well as flow fraction values for terminating arterial branches. Furthermore, details of the benchmark comparison and the pulse wave propagation (PWP) model used herein are provided.
(PDF)

S1 Dataset Comma-separated values file containing pressure data as simulated in the benchmark comparison. This file provides pressure data at thirteen locations, generated using on the one hand the TL model and on the other hand the PWP model. Each column contains a pressure waveform for a specific location and model. Column headers are coded as ‘Seg#_Model_Flow’. For example, ‘Seg1p2_TL_Flow’ denotes the pressure over time for the ‘1+2’ tube as generated by the TL model.
(CSV)

S2 Dataset Comma-separated values file containing flow data as simulated in the benchmark comparison. This file provides flow data at thirteen locations, generated using on the one hand the TL model and on the other hand the PWP model. Each column contains a flow waveform for a specific location and model. Column headers are coded as ‘Seg#_Model_Flow’. For example, ‘Seg1p2_PWP_Flow’ denotes the pressure over time for the ‘1+2’ tube as generated by the PWP model.
(CSV)
