# Supplementary material for: Large vessels as a tree of transmission lines incorporated in the CircAdapt whole-heart model: A computational tool to examine heart-vessel interaction
Source: PLoS Comput Biol. 2019 Jul 15;15(7):e1007173. doi: 10.1371/journal.pcbi.1007173 (PMC6677326; doi:10.1371/journal.pcbi.1007173)
Supplement: S1 Text — This document contains a detailed overview of the transmission line (TL) model’s solving strategy, geometrical- and mechanical properties of arterial- and venous segments belonging to the modelled vascular trees, as well as flow fraction values for terminating arterial branches. Furthermore, details of the benchmark comparison and the pulse wave propagation (PWP) model used herein are provided. (PDF) [file pcbi.1007173.s001.pdf]

S1 Text

Large vessels as a tree of transmission lines incorporated in the CircAdapt whole-circulation model: a computational tool to examine heart-vessel interaction

Maarten H.G. Heusinkveld *[1], Wouter Huberts[1], Joost Lumens[1], Theo Arts[1], Tammo Delhaas[1], and Koen D. Reesink[1]

[1]CARIM School for Cardiovascular Diseases, Maastricht University, NL

*Address correspondence to M.H.G. Heusinkveld
Universiteitssingel 50, room 3.358, 6229 ER Maastricht
E-mail: maarten@heusinkveld.nu

Vascular module

Solving strategy

Pressure and flow in a tube constitutes of the summation of a backward (bw) and a forward (fw) wave component [2]. Therefore, at any location in the tube, it holds that

$$p(z,t) = p_{\mathrm{fw}}(z-ct) + p_{\mathrm{bw}}(z+ct) \ , \tag{S1}$$
$$q(z,t) = \frac{p_{\mathrm{fw}}(z-ct)}{Z} - \frac{p_{\mathrm{bw}}(z+ct)}{Z} \ ,$$

with Z and c the characteristic Womersley number-dependent wave impedance and wave speed defined $Z = \sqrt{L(\alpha_0)/C}$ and $c = 1/\sqrt{L(\alpha_0)C}$, respectively (Section 'Derivation of the attenuation constant, wave speed and wave impedance'). Considering the proximal inlet of a tube, we assume a linear relation between pressure $p_{\mathrm{in}} = p(0,t)$ and flow $q_{\mathrm{in}} = q(0,t)$, characterised by a zero-flow pressure p_{s} and an input resistance Z_{in}:

$$p_{\mathrm{in}} = p_{\mathrm{s}} + Z_{\mathrm{in}} q_{\mathrm{in}} \ . \tag{S2}$$

We derive in Section 'Derivation of source pressure' that quantities p_{s} and Z_{in} can be calculated from p_{bw} and Z using the expressions given by

$$p_{\mathrm{s}} = 2p_{\mathrm{bw}} \ , \tag{S3}$$
$$Z_{\mathrm{in}} = Z \ . \tag{S4}$$

The proximal load relates p_{in} to q_{in}. Using Eq. S2, p_{in} and q_{in} are calculated. Then, for further calculations we need to determine the amplitude of reflected wave p_{fw} at the entrance. It holds

$$p_{\mathrm{fw}} = \frac{1}{2} p_{\mathrm{s}} + Z q_{\mathrm{in}} \ . \tag{S5}$$

A tube, representing a blood vessel with length l, has a proximal and a distal side (Fig. A).

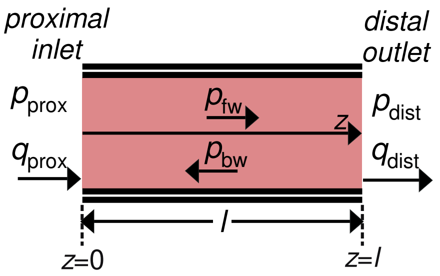

Figure A: Overview of wave propagation in a tube segment, directed along the z-axis. See text for description of symbols.

For each side Eqs. S1 to S5 may be applied with the remark, that flow at the distal side is directed outward, implying that the terms with flow $q_{\mathrm{out}} = q(l,t)$ have to change sign. At each moment in time, the amplitudes of the waves entering on both sides of the

tube are calculated according to Eq. S5. This wave will arrive with a delay τ later at the opposite ending. As derived by Boucher and Kitsios [2] and applied by Krus et al. [6], the contribution of wave attenuation due to friction may be lumped at the proximal and distal sides of the transmission line. Thus, for the proximal side it holds

$$p_{\mathrm{bw,prox}} = p_{\mathrm{bw,dist}}(t-\tau)\exp(-\zeta l), \text{ with } \tau = \frac{l}{c} \,. \tag{S6}$$

Symbols ζ and τ represent the attenuation constant and wave delay time, respectively. Attenuation constant ζ is assumed a function of the characteristic Womersley number (see Section Derivation of the attenuation constant, wave speed and wave impedance). Since $p_{\mathrm{bw,dist}}$ is calculated before, and τ is assumed to be known, $p_{\mathrm{bw,prox}}$ can be calculated. Pressure $p_{\mathrm{fw,dist}}$ is to be calculated similarly. When knowing these wave amplitudes, zero-flow pressure p_{s} and an input resistance Z_{in} can be calculated on both sides of the tube, using Eqs. S3 and S4 and their equivalents at the distal side. Pressures and flows related to wave propagation are superimposed on DC-components of flow q_{DC} and pressure p_{DC} [2]. Furthermore, for small blood vessels, the Poiseuille resistance may become important. For the DC-pressure difference over the tube, related to q_{DC} it holds

$$\Delta p_{\mathrm{DC}} = q_{\mathrm{DC}} \frac{8\pi\eta l}{A^2} \,. \tag{S7}$$

Flow q_{DC}, derived in the online supplement section 'Derivation of the DC-flow', may vary slowly, practically not influencing the behaviour of the wave. Thus, besides the wave, a tube may be subject to a (nearly) constant flow q_{DC}, not being subject to wave attenuation. Since wave impedance Z is known, we may describe the zero-flow pressures $\tilde{p}_{\mathrm{s,prox}}$ and $\tilde{p}_{\mathrm{s,dist}}$ by adding a DC-component to the zero-flow wave pressures ($p_{\mathrm{s,prox}}$ and $p_{\mathrm{s,dist}}$, respectively) as calculated using Eq. S3:

$$\begin{aligned} \tilde{p}_{\mathrm{s,prox}} &= p_{\mathrm{s,prox}} - q_{\mathrm{DC}} Z + \frac{\Delta p_{\mathrm{DC}}}{2} \,, \\ \tilde{p}_{\mathrm{s,dist}} &= p_{\mathrm{s,dist}} + q_{\mathrm{DC}} Z - \frac{\Delta p_{\mathrm{DC}}}{2} \,. \end{aligned} \tag{S8}$$

For solving the differential equations, the solution inside is not needed; between nodes just a phase-delay and damping are imposed. However, if needed, a solution may be computed using the right- and left-ward traveling waves, attenuation constant, wave velocity, and position in the segment. Practically, if pressure and flow waveforms half-way would be required as simulation output, then the tube of interest may be subdivided into two separate tubes.

Around junctions and bifurcations, we assumed continuity of static pressure and balance of mass.

Derivation of the attenuation constant, wave speed and wave impedance

Previously, Huberts et al. [4] showed that for a time-domain analysis, the momentum balance equation can be represented by a lumped-parameter model consisting of a characteristic Womersley number-dependent inertance per unit length ($L(\alpha_0)$) element in series

with a characteristic Womersley number-dependent resistor per unit length ($R(\alpha_0)$) element. We define the characteristic Womersley number (α_0) as $\alpha_0 = r_0\sqrt{\rho\omega_0/\eta}$, with ω_0 the characteristic angular frequency, r_0 the reference lumen radius, and constants ρ and η the blood density and blood dynamic viscosity, respectively. For our time-domain approach, we adopted the method of Huberts et al.[4]. Expressions for $R(\alpha_0)$ and $L(\alpha_0)$ are given by

$$L(\alpha_0) = \overbrace{\left(\frac{1}{2 - c_p(\alpha_0)}\right)}^{g(\alpha_0)} L_0 \ , \tag{S9}$$

$$R(\alpha_0) = \overbrace{\left(\frac{c_q(\alpha_0)}{2 - c_p(\alpha_0)}\right)}^{h(\alpha_0)} R_0 \ . \tag{S10}$$

Here, inertance per unit length (L_0) and Poiseuille resistance per unit length (R_0) are defined $L_0 = \rho/A_0$ and $R_0 = 8\pi\eta/A_0^2$, respectively. The coefficients c_p and c_q were derived by Bessems et al. [1]:

$$c_p(\alpha_0) = \begin{cases} 1 + \frac{\sqrt{2}}{\alpha_0}\left(1 - \frac{\sqrt{2}}{2\alpha_0}\right) & \text{if } \alpha_0 > \sqrt{2} \\ \frac{3}{2} & \text{if } \alpha_0 \leq \sqrt{2} \ , \end{cases}$$
$$c_q(\alpha_0) = \begin{cases} \frac{\alpha_0}{4\sqrt{2}}\left(1 - \frac{\sqrt{2}}{2\alpha_0}\right)^{-1} & \text{if } \alpha_0 > \sqrt{2} \\ \frac{1}{2} & \text{if } \alpha_0 \leq \sqrt{2} \ . \end{cases} \tag{S11}$$

In Fig. B, g and h are depicted as a function of α_0. The ranges chosen for α_0, i.e. $[0, \sqrt{2}]$ and $[\sqrt{2}, \infty]$ render that for the limiting cases of $\alpha_0 = 0$ and $\alpha_0 \to \infty$, the friction term coincides with Womersley theory [1]. Using the expressions for $R(\alpha_0)$ and $L(\alpha_0)$, attenuation

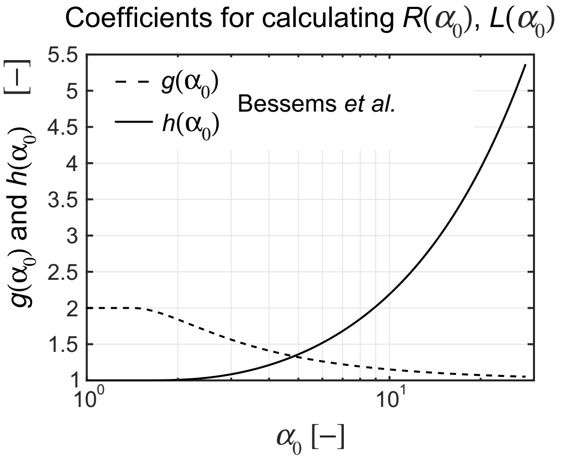

Figure B: Coefficients g and h as a function of the characteristic Womersley number (α_0), used for calculating the characteristic Womersley number-dependent resistance $R(\alpha_0)$ and inertance $L(\alpha_0)$, respectively.

constant ζ is obtained by calculating propagation constant γ, evaluated for characteristic

frequency ω_0. We approximated γ following Matick [7]:

$$\begin{aligned}\gamma = \zeta + j\beta = j\omega\sqrt{L(\alpha_0)C}&\sqrt{1-\frac{jR(\alpha_0)}{\omega L(\alpha_0)}} \\ \approx j\omega\sqrt{L(\alpha_0)C}&\left(1-\frac{jR(\alpha_0)}{2\omega L(\alpha_0)}+\mathcal{O}\left(\frac{1}{\omega^2}\right)\right) \\ \approx \frac{R(\alpha_0)}{2\sqrt{L(\alpha_0)/C}}&+j\omega\sqrt{L(\alpha_0)C}\ ,\end{aligned} \tag{S12}$$

with ζ the attenuation constant per unit length, β the phase constant per unit length and C the compliance per unit length. The characteristic Womersley number-dependent wave speed c was calculated using

$$c = \frac{\omega}{\beta} = \frac{1}{\sqrt{L(\alpha_0)C}}\ . \tag{S13}$$

Finally, Womersley number-dependent wave impedance was calculated using

$$Z = \sqrt{\frac{L(\alpha_0)}{C}}\ . \tag{S14}$$

Derivation of source pressure

As previously described, it holds for pressure and flow at any location that

$$\begin{aligned} p(z,t) &= p_{\text{fw}}(z-ct) + p_{\text{bw}}(z+ct)\ , \\ q(z,t) &= \frac{p_{\text{fw}}(z-ct)}{Z} - \frac{p_{\text{bw}}(z+ct)}{Z}\ . \end{aligned} \tag{S15}$$

Pressure and flow at the proximal side of the tube are given by

$$\begin{aligned} p_{\text{prox}} &= p_{\text{fw,prox}} + p_{\text{bw,prox}}\ , \\ q_{\text{prox}} &= \frac{p_{\text{fw,prox}}}{Z_{\text{prox}}} - \frac{p_{\text{bw,prox}}}{Z_{\text{prox}}}\ . \end{aligned} \tag{S16}$$

If $q_{\text{prox}} = 0$ and $p_{\text{bw,prox}}$ is known, then for proximal source pressure $p_{\text{s,prox}} = p_{\text{prox}}$ it follows after substitution of Eq. S16:

$$p_{\text{s,prox}} = 2p_{\text{bw,prox}}\ . \tag{S17}$$

For short circuit flow $q_{\text{s,prox}} = q_{\text{prox}}$ with $p_{\text{prox}} = 0$, it follows after substitution of Eq. S16:

$$q_{\text{s,prox}} = \frac{2p_{\text{bw,prox}}}{Z_{\text{prox}}}\ . \tag{S18}$$

The source impedance of the proximal side of the tube is calculated using

$$Z_{\text{prox}} = \frac{p_{\text{s,prox}}}{q_{\text{s,prox}}} = Z\ . \tag{S19}$$

Finally, the applied equations for the distal side of the tube are given by

$$p_{\mathrm{s,dist}} = 2p_{\mathrm{fw,dist}} \;, \qquad \text{(S20)}$$
$$q_{\mathrm{s,dist}} = \frac{2p_{\mathrm{fw,dist}}}{Z_{\mathrm{dist}}} \;,$$
$$Z_{\mathrm{dist}} = \frac{p_{\mathrm{s,dist}}}{q_{\mathrm{s,dist}}} = Z \;.$$

Derivation of the DC-flow

The DC-flow (q_{DC}), averaged over the tube length, changes due to contribution of flow waves entering and leaving from the proximal and distal side. In case no wave enters, the waves inside the tube will disappear completely after wave delay time τ. Using Eq. S8, reaching of the DC-steady state (i.e. term B of Eq. S21 becoming zero) was approximated by a first order differential equation with time constant equal to τ:

$$\frac{dq_{\mathrm{DC}}}{dt} = \overbrace{\left(\frac{p_{\mathrm{s,dist}} - p_{\mathrm{s,prox}}}{2Z} - q_{\mathrm{DC}}\right)}^{B} \frac{1}{\tau} \;. \qquad \text{(S21)}$$

Benchmark comparison between TL and PWP model

For the benchmark comparison, we implemented a network of tubes describing the central arteries (i.e. the aorta, carotid artery, and vertebral artery), as well as the arteries of the left arm (Fig. C). Geometrical and mechanical properties of all tubes were based on Reymond et al. [10], (Table B). Vessel stiffness coefficient (k) for the aortic segments, vertebral artery and carotid artery was estimated by fitting the constitutive law to *in vitro* data from Hayashi et al. [3]. For the radial, ulnar and interosseous arteries, stiffness was assumed equal to vertebral artery stiffness. For the subclavian–axillary–brachial arterial segment, stiffness was taken as the mean of aortic and carotid artery stiffness (Table B). Flow fractions were based on previously published patient measurements with duplex ultrasound and phase-contrast magnetic resonance imaging [4]. For the benchmark comparison, we defined a half-sinusoidal inflow profile as a proximal inlet boundary condition ($q_{\mathrm{inflow}}(t)$):

$$q_{\mathrm{inflow}}(t) = \begin{cases} q_{\mathrm{p}} \sin\left(\frac{\pi t}{t_{\mathrm{c}}}\right) & \text{if } 0 \leq t \leq t_{\mathrm{c}} \\ 0 & \text{if } t > t_{\mathrm{c}} \;. \end{cases} \qquad \text{(S22)}$$

Distal tubes were terminated using 3WKs. For these simulations, time step (Δt) was kept at 0.5 ms, duty cycle (t_{c}) at 0.3 s, cardiac cycle duration (T) at 0.85 s, and peak flow rate (q_{p}) at 350 ml s^{-1}, resulting in a physiological cardiac output. Boundary conditions were kept equal for the TL model and PWP model. Reference pressure (p_0) was kept at 105 mmHg, external pressure (p_{ext}) at 0 mmHg and outflow pressure at 4 mmHg. We chose an element size of 0.01 m. Convergence of the calculated haemodynamics was evaluated after each simulated cardiac cycle. Hereto, the calculated nodal pressures, $p(t)$, of the

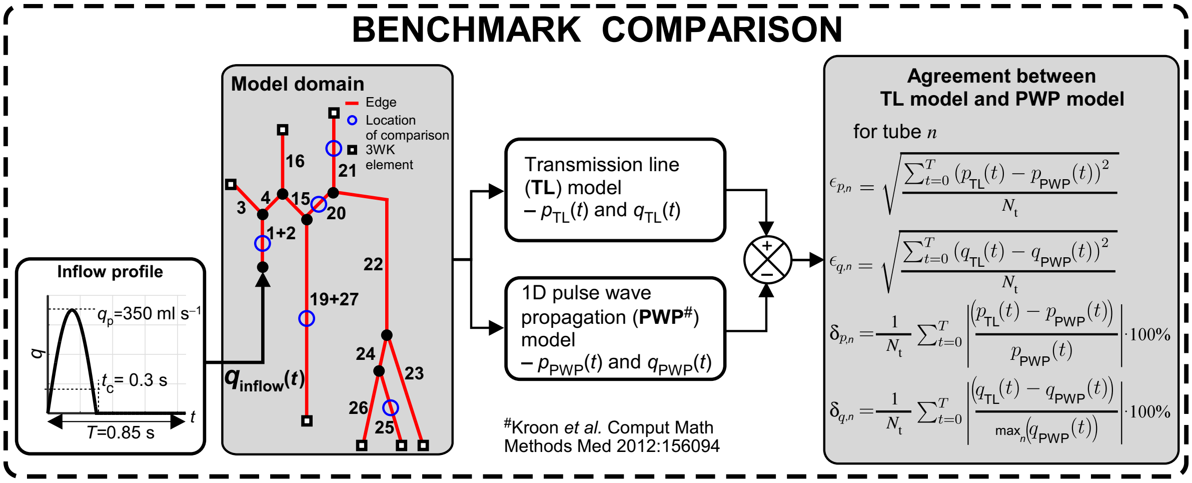

Figure C: Benchmark comparison of the transmission line (TL) model with the previously validated 1D pulse wave propagation (PWP) model. We implemented a network of 13 tubes representing the large central arteries and the arteries of the left arm. Geometrical and mechanical properties of arterial segments that were used in the benchmark comparison are given in Table B. A half-sinusoidal inflow profile (q_{inflow}) was prescribed to the ascending aorta. Boundary conditions; proximal inflow, termination of distal vessels using a non-linear three element winkessel model (3WK), vessel geometry and material properties were kept the same for the TL model and PWP model. Evaluated agreement metrics were root mean square errors ϵ_p and ϵ_q, as well as the relative errors δ_p and δ_q. Furthermore, N_{t} denotes the number of time points for which a comparison was made. A graphical comparison between pressure and flow waveforms is given for five tubes (blue circles).

current cycle (i_{cyc}) were compared to those of the previous cycle ($i_{\text{cyc}} - 1$). An expression for the haemodynamics convergence norm (ϵ_{norm}) is given as a relative root mean square:

$$\epsilon_{\text{norm}} = \max_n \left(\sqrt{\frac{\sum_{t=0}^{T} \left(p_{n,i_{\text{cyc}}}^{t} - p_{n,i_{\text{cyc}}-1}^{t} \right)^2}{\sum_{t=0}^{T} \left(p_{n,i_{\text{cyc}}-1}^{t} \right)^2}} \right), \tag{S23}$$

with n referring to the tube number as given in Table B. Throughout simulations, the haemodynamics convergence criterion was kept at 10^{-2}. Agreement between p and q waveforms, expressed by root mean square errors ϵ_p and ϵ_q, as well as relative errors δ_p and δ_q, was calculated for the center element of each tube (Fig. C).

Numerical implementation of the pulse wave propagation model of Kroon et al. [5]

For the 1D vessels of the PWP model, we assumed the same constitutive law as used for the TL model. Moreover for the periphery, we implemented the same non-linear three-element windkessel as used for the TL model. Coupling and solving of 1D mass conservation and momentum balance equations (after neglecting the convective acceleration term) and the 0D equation for pressure and flow in the periphery was performed using the method previously published by Kroon et al. [5]. We used a simplified trapezoidal scheme

for spatial discretisation. The second-order backward difference scheme was used for time discretisation.

Simulations performed using the CircAdapt–3WK model

As described in the main text of this article, we additionally performed reference- and hypertension simulations using the CircAdapt model with the systemic circulation lumped into the non-linear three-element windkessel model (REF–3WK and HYP–3WK, respectively). For the REF–3WK simulation, reference pressure was 105 mmHg and vessel stiffness coefficient (k) was kept equal to 8 for the arterial side and 10 for the venous side, respectively. For the HYP–3WK simulation, reference pressure was increased to 135 mmHg and the k-value of the systemic arterial 3WK was set to 14. For both simulations, characteristic vessel bed length (l_{AV}) was chosen 0.8 m [11].

Code availability and calculation loop of the CircAdapt–TL model

The CircAdapt–TL model source code can be retrieved from `https://github.com/Mheu1991/CircAdaptTL/`. Table A refers to specific lines of MATLAB-code providing programmatic logic of the aforementioned relations (i.e. Eq S#) that were implemented to solve our TL model. Furthermore, Fig. D illustrates the calculation loop in CircAdapt–TL.

Table A: Index linking mathematical relations of the transmission line model to MATLAB-scripts and underlying lines of code. Previous publications comprising existing CircAdapt modules are indicated.

Equation	MATLAB-script	Line no.
S5	`PNodeVDot.m`	52
S6	`TubeDelays.m`	36, 37, 85, 86
S7	`TubeV2p.m`	51, 52
S8	`TubeDelays.m`	60 to 63
S9	`CalcApproximateVelocityProfile.m`	29
S10	`CalcApproximateVelocityProfile.m`	30
S11	`CalcApproximateVelocityProfile.m`	27, 28
S12	`CalcApproximateVelocityProfile.m`	36
S13	`CalcApproximateVelocityProfile.m`	37
S14	`CalcApproximateVelocityProfile.m`	38
S21	`TubeDelays.m`	57 to 59

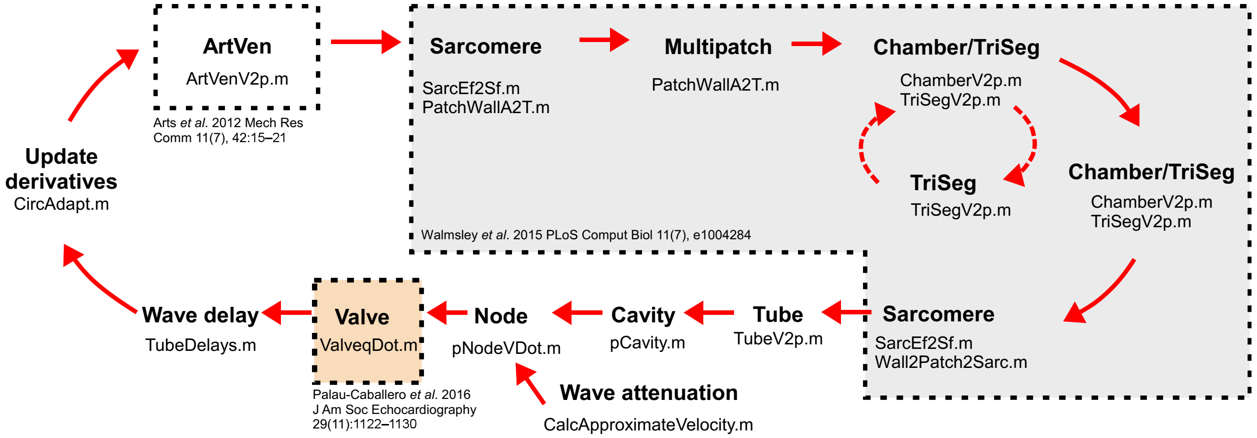

Figure D: Calculation loop of the CircAdapt–TL model.

Calculation of wave intensity

The fundamental basis of wave intensity analysis is described in Parker [9]. Briefly, at a fixed measurement location, waves are considered incremental fronts for which the sum of successive forward- or backward travelling wavefronts form the measured pressure- or flow velocity waveform. Wave intensity analysis determines the magnitude and direction of wavefronts from derivatives of local pressure and flow velocity [9]. Wave intensity $(\mathrm{d}I)$ is here calculated using the time derivatives of pressure $(\mathrm{d}p/\mathrm{d}t)$ and flow velocity $(\mathrm{d}U/\mathrm{d}t)$:

$$\mathrm{d}I = \frac{\mathrm{d}p}{\mathrm{d}t}\frac{\mathrm{d}U}{\mathrm{d}t} \; . \tag{S24}$$

Wave intensity is positive when forward travelling waves are dominant, whereas it is negative when backward travelling waves are dominant. Wave intensity itself may also be calculated for forward- and backward waves. This requires separation of the simulated pressure and flow velocity waveforms into their respective forward- and backward components. By assuming balance of mass and momentum in elastic tubes and neglecting viscous friction, a simple relation between the time derivatives of pressure and flow velocity is given by [9]

$$\frac{\mathrm{d}p}{\mathrm{d}t} = \rho c \frac{\mathrm{d}U}{\mathrm{d}t} \; , \tag{S25}$$

with c the local pulse wave velocity. Parker [9] showed that the relation as given in Eq. S25 may be written for forward- and backward components as

$$\begin{aligned} \frac{\mathrm{d}p^+}{\mathrm{d}t} &= \rho c \frac{\mathrm{d}U^+}{\mathrm{d}t} \; , \text{and} \\ \frac{\mathrm{d}p^-}{\mathrm{d}t} &= -\rho c \frac{\mathrm{d}U^-}{\mathrm{d}t} \; , \end{aligned} \tag{S26}$$

with superscripts "$+$", and "$-$" the forward- and backward wave components. Because we assumed that summation of backward- and forward waves yields the measured pressure

and flow velocity waveforms, the aforementioned equation may be expressed as [9]

$$\begin{aligned}\frac{\mathrm{d}p^+}{\mathrm{d}t} &= \left(\frac{\mathrm{d}p}{\mathrm{d}t} + \rho c \frac{\mathrm{d}U}{\mathrm{d}t}\right)/2\ , \\ \frac{\mathrm{d}p^-}{\mathrm{d}t} &= \left(\frac{\mathrm{d}p}{\mathrm{d}t} - \rho c \frac{\mathrm{d}U}{\mathrm{d}t}\right)/2\ , \\ \frac{\mathrm{d}U^+}{\mathrm{d}t} &= \left(\frac{\mathrm{d}U}{\mathrm{d}t} + \frac{\mathrm{d}p}{\mathrm{d}t}/\rho c\right)/2\ , \text{and} \\ \frac{\mathrm{d}U^-}{\mathrm{d}t} &= \left(\frac{\mathrm{d}U}{\mathrm{d}t} - \frac{\mathrm{d}p}{\mathrm{d}t}/\rho c\right)/2\ ,\end{aligned} \tag{S27}$$

Using Eq. S24, intensity for forward- and backward waves, and the net pressure wave is expressed by

$$\begin{aligned}\mathrm{d}I^+ &= \frac{\mathrm{d}p^+}{\mathrm{d}t}\frac{\mathrm{d}U^+}{\mathrm{d}t}\ , \\ \mathrm{d}I^- &= \frac{\mathrm{d}p^-}{\mathrm{d}t}\frac{\mathrm{d}U^-}{\mathrm{d}t}\ , \text{and} \\ \mathrm{d}I &= \mathrm{d}I^+ + \mathrm{d}I^-\ .\end{aligned} \tag{S28}$$

For mathematical derivation of Eqs. S27 and S28, we refer to the work of Parker [9]. It should be noted that whereas Parker [9] calculated wave intensity based on *instantaneous differences* in pressure and flow velocity (i.e. $\mathrm{d}p$ and $\mathrm{d}U$), we calculated *time derivatives* (i.e. $\mathrm{d}p/\mathrm{d}t$ and $\mathrm{d}U/\mathrm{d}t$). As a result, wave intensity is given in units of [W m^{-2} s^{-2}], instead of in [W m^{-2}].

Description of geometrical and mechanical parameters of the implemented network of arteries and veins

Table B: Geometrical and mechanical properties of arterial segments, based on Reymond et al. [10]. The italic q_{AV} values represent the terminal flow (here expressed as a percentage of cardiac output) as used in the benchmark comparison. Tube numbers in squares (e.g. [3]) refer to segments that were also used in the benchmark comparison.

tube #	name [-]	l [m]	d_{prox} [10^{-3} m]	d_{dist} [10^{-3}m]	k [-]	q_{AV} [% of CO]
[1]	ascending aorta 1	0.020	29.4	29.3	10	
[2]	ascending aorta 2	0.020	29.3	28.8	10	
[3]	brachiocephalic artery	0.040	20.2	18.0	10	*20*
[4]	aortic arch A	0.020	25.1	24.0	10	
5	r. subclavian artery	0.040	11.5	9.0	10	
6	r. common carotid artery	0.100	13.5	7.0	14	
7	r. vertebral artery	0.150	3.7	2.8	16	5
8	r. subcl. B + axill. + brach.	0.430	8.1	4.7	12	
9	r. radial artery	0.240	3.7	3.1	12	2.3
10	r. ulnar artery A	0.070	3.7	3.4	16	
11	r. interosseous artery	0.080	2.1	1.8	16	0.14
12	r. ulnar artery B	0.180	3.2	2.8	16	2.3
13	r. internal carotid artery	0.180	5.7	4.3	16	6
14	r. external carotid artery	0.050	5.0	4.5	14	2
[15]	aortic arch B	0.040	21.4	20.8	14	
[16]	l. common carotid artery	0.140	12.0	6.0	10	*15*
17	l. internal carotid artery	0.180	5.3	4.1	14	6
18	l. external carotid artery	0.050	4.7	4.3	14	2
[19]	thoracic aorta A	0.060	20.0	18.9	10	
[20]	l. subclavian artery A	0.040	11.0	8.5	12	
[21]	l. vertebral artery	0.150	3.7	2.8	16	5, *5*
[22]	l. subcl. B + axill. + brach.	0.430	8.1	4.7	10	
[23]	l. radial artery	0.240	3.5	2.8	16	2.3, *2*
[24]	l. ulnar artery A	0.070	4.3	4.3	16	
[25]	l. interosseous artery	0.079	1.8	1.8	16	0.14, *2*
[26]	l. ulnar artery B	0.180	4.1	3.7	16	2.3, *2*
[27]	thoracic aorta B	0.110	16.5	12.9	10	*54*
28	abdominal aorta A	0.060	12.2	12.2	10	
29	celiac artery	0.040	7.8	6.9	12	
30	hepatic artery	0.070	5.4	4.4	12	3.3
31	gastric artery	0.080	3.2	3.0	12	2.2
32	splenic artery	0.070	4.2	3.9	12	5.2
33	superior mesenteric artery	0.060	7.9	7.1	12	13
34	abdominal aorta B	0.020	11.5	11.8	10	

35	l. renal artery	0.040	5.2	5.2	10	10.7
36	abdominal aorta C	0.020	11.8	11.0	10	
37	r. renal artery	0.040	5.2	5.2	12	10.7
38	abdominal aorta D	0.110	11.6	7.0	10	
39	inferior mesenteric artery	0.050	4.7	3.2	12	1.8
40	abdominal aorta E	0.020	10.8	6.1	10	
41	r. common iliac artery	0.060	7.9	4.0	16	
42	l. common iliac artery	0.060	7.9	4.0	16	
43	l. external iliac artery	0.150	6.4	3.7	16	
44	l. internal iliac artery	0.050	4.0	2.8	16	1.5
45	l. femoral artery	0.443	5.2	2.3	30	
46	l. deep femoral artery	0.126	4.0	3.7	30	1.5
47	l. posterior tibial artery	0.321	3.1	2.8	30	2.5
48	l. anterior tibial artery	0.343	2.6	2.3	30	2.5
49	r. external iliac artery	0.144	6.4	3.7	30	
50	r. internal iliac artery	0.050	4.0	2.8	30	2.5
51	r. femoral artery	0.450	5.2	2.3	30	
52	r. deep femoral artery	0.130	4.0	3.7	30	2.1
53	r. posterior tibial artery	0.330	3.1	2.8	30	2.5
54	r. anterior tibial artery	0.350	2.6	2.3	30	2.2

Table C: Geometrical and mechanical properties of venous segments, based on Müller and Toro [8].

tube #	name	l [m]	d_{prox} $[10^{-3}$m]	d_{dist} $[10^{-3}$m]	k [-]
55	vena cava superior	0.035	16.0	16.0	10
56	r. brachiocephalic vein	0.040	11.3	11.3	10
57	r. internal carotid vein	0.025	5.0	5.0	10
58	r. external carotid vein	0.025	5.0	5.0	10
59	r. subclavian vein A	0.030	11.3	11.3	10
60	r. vertebral vein	0.110	3.2	3.2	10
61	r. subclavian vein B	0.030	10.4	10.4	10
62	r. subclavian vein C	0.270	10.4	10.4	10
63	r. radial vein	0.406	4.0	4.0	10
64	r. ulnar vein A	0.100	4.0	4.0	10
65	r. interosseous vein	0.070	2.0	2.0	10
66	r. ulnar vein B	0.306	4.0	4.0	10
67	l. brachiocephalic vein	0.075	10.9	10.9	10
68	l. internal carotid vein	0.025	5.0	5.0	10
69	l. external carotid vein	0.025	5.0	5.0	10
70	l. subclavian vein A	0.030	11.2	11.2	10
71	l. vertebral vein	0.110	3.2	3.2	10
72	l. subclavian vein B	0.030	10.4	10.4	10
73	l. subclavian vein C	0.270	10.4	10.4	10
74	l. radial vein	0.406	4.0	4.0	10
75	l. ulnar vein A	0.100	4.0	4.0	10
76	l. interosseous vein	0.070	2.0	2.0	10
77	l. ulnar vein B	0.306	4.0	4.0	10
78	vena cava inferior	0.020	15.2	15.2	10
79	celiac vein	0.040	9.8	9.8	10
80	gastric vein	0.068	9.8	9.8	10
81	hepatic vein	0.068	9.8	9.8	10
82	splenic vein	0.068	9.8	9.8	10
83	superior mesenteric vein	0.068	9.8	9.8	10
84	vena cava inferior B	0.015	15.2	15.2	10
85	l. renal vein	0.032	5.0	5.0	10
86	r.renal vein	0.032	5.0	5.0	10
87	vena cava inferior C	0.015	15.2	15.2	10
88	vena cava inferior D	0.125	15.2	15.2	10
89	inferior mesenteric vein	0.060	9.0	9.0	10
90	vena cava inferior E	0.080	15.2	15.2	10
91	r. common iliac vein A	0.038	11.6	11.6	10
92	r. common iliac vein B	0.020	11.6	11.6	10
93	r. inner iliac vein	0.050	3.0	3.0	10
94	r. external iliac vein	0.144	6.0	5.0	10
95	r. deep femoral vein	0.126	7.0	7.0	10

96	r. femoral vein	0.254	7.0	7.0	10
97	r. posterior tibial vein	0.173	3.0	3.0	10
98	r. anterior tibial vein	0.173	3.0	3.0	10
99	l. common iliac vein A	0.038	11.6	11.6	10
100	l. common iliac vein B	0.020	11.6	11.6	10
101	l. inner iliac vein	0.050	3.0	3.0	10
102	l. external iliac vein	0.144	10.0	10.0	10
103	l. deep femoral vein	0.126	7.0	7.0	10
104	l. femoral vein	0.254	7.0	7.0	10
105	l. posterior tibial vein	0.173	3.0	3.0	10
106	l. anterior tibial vein	0.173	3.0	3.0	10

References

[1] Bessems D, Rutten M, van de Vosse FN. A wave propagation model of blood flow in large vessels using an approximate velocity profile function. Journal of Fluid Mechanics. 2007;580:145–168.

[2] Boucher R, Kitsios E. Simulation of fluid network dynamics by transmission line modelling. Proceedings of the Institution of Mechanical Engineers, Part C: Journal of Mechanical Engineering Science. 1986;200(1):21–29.

[3] Hayashi K, Handa H, Nagasawa S, Okumura A, Moritake K. Stiffness and elastic behavior of human intracranial and extracranial arteries. Journal of Biomechanics. 1980;13(2):175181–179184.

[4] Huberts W, Bode A, Kroon W, Planken R, Tordoir J, Van de Vosse F, et al. A pulse wave propagation model to support decision-making in vascular access planning in the clinic. Medical Engineering & Physics. 2012;34(2):233–248.

[5] Kroon W, Huberts W, Bosboom M, van de Vosse F. A numerical method of reduced complexity for simulating vascular hemodynamics using coupled 0D lumped and 1D wave propagation models. Computational and Mathematical Methods in Medicine. 2012;2012.

[6] Krus P, Weddfelt K, Palmberg JO. Fast pipeline models for simulation of hydraulic systems. Journal of Dynamic Systems, Measurement, and Control. 1994;116(1):132–136.

[7] Matick RE. Transmission lines for digital and communication networks: an introduction to transmission lines high-frequency and high-speed pulse characteristics and applications. IEEE press; 1995.

[8] Müller LO, Toro EF. A global multiscale mathematical model for the human circulation with emphasis on the venous system. International Journal for Numerical Methods in Biomedical Engineering. 2014;30(7):681–725.

[9] Parker KH. An introduction to wave intensity analysis. Medical & Biological Engineering & Computing. 2009;47(2):175–88.

[10] Reymond P, Merenda F, Perren F, Rüfenacht D, Stergiopulos N. Validation of a one-dimensional model of the systemic arterial tree. American Journal of Physiology-Heart and Circulatory Physiology. 2009;297(1):H208–H222.

[11] Wesseling K, Jansen J, Settels J, Schreuder J. Computation of aortic flow from pressure in humans using a nonlinear, three-element model. Journal of Applied Physiology. 1993;74(5):2566–2573.
